# Overexpression of *MsRCI2A, MsRCI2B*, and *MsRCI2C* in Alfalfa (*Medicago sativ*a L.) Provides Different Extents of Enhanced Alkali and Salt Tolerance Due to Functional Specialization of *MsRCI2s*

**DOI:** 10.3389/fpls.2021.702195

**Published:** 2021-08-19

**Authors:** Chunxin Li, Tingting Song, Lifeng Zhan, Chunlong Cong, Huihui Xu, Li Dong, Hua Cai

**Affiliations:** ^1^College of Life Sciences, Northeast Agricultural University, Harbin, China; ^2^College of Animal Sciences and Technology, Northeast Agricultural University, Harbin, China

**Keywords:** *RCI2* genes, alkali tolerance, salt tolerance, *Medicago saliva* L., gene overexpression

## Abstract

Rare cold-inducible 2/plasma membrane protein 3 (*RCI2/PMP3*) genes are ubiquitous in plants and belong to a multigene family whose members respond to a variety of abiotic stresses by regulating ion homeostasis and stabilizing membranes, thus preventing damage. In this study, the expression of *MsRCI2A*, *MsRCI2B*, and *MsRCI2C* under high-salinity, alkali and ABA treatments was analyzed. The results showed that the expression of *MsRCI2A*, *MsRCI2B*, and *MsRCI2C* in alfalfa (*Medicago sativa* L.) was induced by salt, alkali and ABA treatments, but there were differences between *MsRCI2* gene expression under different treatments. We investigated the functional differences in the MsRCI2A, MsRCI2B, and MsRCI2C proteins in alfalfa (*Medicago sativa* L.) by generating transgenic alfalfa plants that ectopically expressed these *MsRCI2*s under the control of the CaMV35S promoter. The *MsRCI2A/B/C*-overexpressing plants exhibited different degrees of improved phenotypes under high-salinity stress (200 mmol.L^–1^ NaCl) and weak alkali stress (100 mmol.L^–1^ NaHCO_3_, pH 8.5). Salinity stress had a more significant impact on alfalfa than alkali stress. Overexpression of *MsRCI2*s in alfalfa caused the same physiological response to salt stress. However, in response to alkali stress, the three proteins encoded by *MsRCI2*s exhibited functional differences, which were determined not only by their different expression regulation but also by the differences in their regulatory relationship with *MsRCI2*s or *H^+^-ATPase*.

## Introduction

Alfalfa (*Medicago sativa* L.) is an important perennial leguminous plant species that has high nutritional value and high yielding (Yang, 2017; Song et al., 2021). However, extreme environmental conditions, especially soil salinization and alkalization, severely limit the growth and production of alfalfa worldwide (Dong et al., 2014). Therefore, improving salt-alkali tolerance is a priority target trait in alfalfa breeding.

The rare cold-inducible 2 (*RCI2*) gene encodes a highly conserved small-molecule hydrophobic peptide that is ubiquitous in prokaryotes and eukaryotes. The small membrane-localized protein encoded by the *RCI2* gene belongs to the plasma membrane protein 3 (*PMP3*) family, whose members are closely related to the abiotic stress response (Kim et al., 2021) and is similar to Pmp3p in terms of regulating ion homeostasis on the basis of similarities in protein structure and location as well as evidence of RCI2 involvement in regulating Na^+^ and K^+^ levels (Kim et al., 2020). Overexpression of *AtRCI2A* or *MpRCI* in Arabidopsis reduced its Na^+^ content in shoots and increased its K^+^ content in both shoots and roots when growing in high Na^+^ or high K^+^ conditions (Mitsuya et al., 2006; Liu et al., 2012). In addition, many studies have proposed that during cold or salt stress, RCI2s stabilize membranes, thus preventing damage (Kim et al., 2007, 2016; Fu et al., 2012), and exhibit a reduction in the treatment-induced accumulation of H_2_O_2_ and malondialdehyde, water loss and ion leakage.

Long et al. (2015) isolated *MsRCI2A* and *MtRCI2*(*A*-*E*) from *Medicago sativa* L. and *Medicago truncatula* and found that the expression of the *MsRCI2A* and *MtRCI2*(*A*-*D*) genes was highly induced by salt stress. Based on the sequences, the six RCI2 proteins in *M. sativa* and *M. truncatula* can be divided into two groups. MsRCI2A and MtRCI2(A-C) belong to the first group, and MtRCI2(D-E) belong to the second group. The proteins in the first group contain about 54 amino acids, whereas those in the second are comprised of about 76 amino acids, and have extra C-terminal tails of 20 amino acids. Complementation analysis of the ΔPMP3 yeast mutant shows that MsRCI2A and MtRCI2(A-C) are able to complement for the loss of the yeast gene PMP3. Others, overexpression of *MsRCI2A* in Arabidopsis plants resulted in improved salt tolerance. Due to the functional specialization of RCI2 proteins, research on the function of other MsRCI2s is also necessary. In addition, overexpression of *MsRCI2s* in alfalfa was performed to determine the specific function of MsRCI2s, the information of which is beneficial for analyzing the relationships between *MsRCI2* genes and other genes in alfalfa.

An increasing number of studies have shown that adaptive strategies to salt and alkali stresses are quite different and that alkali stress causes greater injury than salt stress (Wang et al., 2012; Huihui et al., 2020). In this study, we attempted to explore whether *MsRCI2A-C* genes involved in salt or alkali stress response and improve the salt or alkali tolerance of alfalfa. In addition, we investigated the different functions of *MsRCI2A-C* in response to salt and alkali stresses. A possible way to overexpress different MsRCI2-like genes for the engineering of salinity and alkali tolerant alfalfa and some other legumes plants has also been suggested.

## Materials and Methods

### Cloning of *MsRCI2* Genes and Multiple Sequence Alignment

According to the coding regions of the target genes *MsRCI2A* (JQ665271), *MsRCI2B* (Medtr7g111450), and *MsRCI2C* (Medtr7g111350), we designed specific primers and used alfalfa cDNA as a template to amplify the target genes via PCR. The primers used are listed in Supplementary Table 1. Multiple sequence alignment of the stem-loop sequence of the RCI2 genes was performed using DNAMAN version 8.0 software (Lynnon Biosoft) with the default parameters. All of the sequences were downloaded from Phytozome version 11.0,^1^ and a phylogenetic tree was constructed by the neighbor-joining (NJ) method via MEGA 6.^2^ All the DNA sequences were converted into amino acid sequences before analysis (involved Gene ID and name see Supplementary Table 2).

### Plant Materials and Growth Conditions

Alfalfa (*M. sativa* “Longmu 806”) was used for alkali, salt, and ABA treatment experiments. The plants were cultivated in a greenhouse at 24 ± 2°C under 16 h of light and 8 h of darkness. Twenty-day-old seedlings were utilized for alkali, salt and ABA treatments. For alkali stress, the plants were treated with 100 or 200 mmol.L^–1^ NaHCO_3_ (pH 8.5), and leaves were harvested from the seedlings at 0, 1, 2, 5, 10, 24, and 48 h after treatment. The collected samples were immediately frozen and stored at -80∘C for total RNA extraction. With respect to salt stress, plants were treated with 200 mmol.L^–1^ NaCl, and the samples were harvested in the same manner as those for the alkali stress treatments. For ABA treatment, plants were treated with 100 μmol.L^–1^ ABA, and samples were taken at 0, 2, 4, 8, 12, 24, and 48 h after treatment. For all of the above samples, three biological replicates were included for each sample.

### RNA Extraction and qRT-PCR Analysis

To measure the mRNA expression level of *MsRCI2*s after abiotic stress treatment, total RNA was extracted using an RNeasy Plant Mini Kit (CW Biotech, Beijing, China). The RNA was then reverse transcribed into cDNA for use as a template. The quality of the cDNA was assessed via PCR using *GAPDH*-specific primers. Quantitative real-time PCR was carried out in a 96-well (10 μL) system using Trans Start Top Green qPCR SuperMix (Vazyme Biotech, Nanjing, China); *GAPDH* was used as an internal standard. All of the reactions were performed in biological triplicates with the use of RNA samples extracted from three independent plant materials. The expression level of each gene from 1 to 48 h was compared with that at 0 h; the expression was estimated by the 2^–ΔΔCt^ method. The primers used are listed in Supplementary Table 1.

### Generation of *MsRCI2*-Overexpressing Transgenic Plants

To develop *MsRCI2* overexpression constructs, the coding sequences of *MsRCI2*s were cloned from the cDNA of alfalfa. The PCR products were then inserted into a pMDC123 vector, in which the genes were fused to the cauliflower mosaic virus 35S RNA promoter (CaMV35S); the vector carried a *Bar* resistance gene that affords insensitivity to the herbicide glufosinate (Supplementary Figure 3A). The *pMDC123-MsRCI2* vectors were then electroporated into *Agrobacterium tumefaciens* strain LBA4404.

For transformation, the cotyledonary nodes were cultured from germinated seeds of Longmu 806; these cotyledonary nodes were used as transformation receptors (Sun et al., 2016), followed by subculture, rooting and domestication. Briefly, the transformants were selected by the use of 1.0 mg.L^–1^ glufosinate ammonium, and regenerated shoots were rooted on 1/2-strength Murashige and Skoog media. Finally, the glufosinate-positive seedlings were transplanted into soil and grown in a greenhouse under controlled conditions.

To screen the transgenic lines, genomic DNA from young leaves of glufosinate-resistant plants was extracted according to the cetyltrimethylammonium bromide protocol (Permingeat et al., 1998). The total DNA of each strain was used as a template, and PCR-based detection was performed with the use of primers specific to the *Bar* gene (Supplementary Table 1).

PCR-positive plants were further confirmed, and the expression level of the *MsRCI2*s in the transgenic alfalfa plants was measured via semiquantitative RT-PCR and quantitative real-time PCR. Total RNA extraction and first-strand cDNA synthesis were performed as described above. We subsequently propagated these plants, and their phenotypes were scored, after which they were subjected to molecular and physiological analysis.

### Alkali and Salt Stress Treatments

An asexual cutting propagation method was used to propagate the WT alfalfa plants and transgenic alfalfa plants. Uniform transgenic and wild-type (WT) alfalfa plants were separately transplanted into cylindrical plastic pots that contained vermiculite and perlite (1:1) at 25°C under a 16/8 h (light/dark) photoperiod and 50% relative humidity (RH). The pots were then placed in rectangular plastic trays. The plants were watered every 2 days with 1/5-strength Hoagland nutrient solution for 20 days; thereafter, alfalfa plants displaying the same growth were selected for the control group and other treatment groups, including the alkali (100 mmol.L^–1^ NaHCO_3_, pH 8.5) and salt (200 mmol.L^–1^ NaCl) groups. The absorbability of each pot was still calculated as 200 mL, and lye was poured into the bottom of the large tray that contained the pots. In the control group and treatment group, samples were taken at 0, 6, and 12 days after the beginning of the treatment for physio-biochemical analysis.

### Physio-Biochemical Analysis of Transgenic Plants

The determinations of physiological and biochemical indexes were divided into three parts. First, the chlorophyll (Chl) content of leaves was measured using a SPAD chlorophyll meter; each recorded value involved 3 biological replicates and 10 technical replicates (the average of which was used for each individual plant) (Sun et al., 2020; Song et al., 2021). The relative conductivity of a leaf blade was then determined by the vacuum method. Afterward, the malondialdehyde (MDA) content was measured by the use of a Solarbio kit (Beijing Solarbio Science Technology Co., Ltd., Beijing, China) and then measured by a UV-visible spectrophotometer. The MDA content was calculated as the difference between the absorbance at 532 and 600 nm. Second, the activities of antioxidant enzymes in the WT and transgenic plants were measured after abiotic stress. The superoxide dismutase (SOD), catalase (CAT), and peroxidase (POD) activities were measured with a Solarbio kit (Beijing Solarbio Science Technology Co., Ltd.), and the optical density was read at 560, 240, and 470 nm. Finally, the contents of proline and soluble sugars in the transgenic plants and WT plants were measured after abiotic stress with a Solarbio kit (Beijing Solarbio Science Technology Co., Ltd.), and the optical density was read at 520 and 620 nm.

### Analysis of Expression Patterns of Related Genes

The protein encoded by *MsRCI2* can regulate ion homeostasis. Whether there is a mutual regulatory relationship between members of *RCI2* gene families also needs to be determined. To verify the changes in the expression of related genes, including *H^+^-ATPase* and *MsRCI2A/B/C*, qPCR was used to measure the gene expression differences in the leaves of transgenic and WT plants after alkali treatment. Total RNA extraction and first-strand cDNA synthesis were performed as described above. The expression level of each gene after 12 h of alkali treatment was compared with that at 0 h. The primers used are listed in Supplementary Table 1.

### Statistical Analysis

Three biological replicates were assessed for each group, and the test data were analyzed via Microsoft Excel 2010. Significant differences were analyzed with GraphPad Prism 9.0 via one-way ANOVA or Student’s *t*-test. *P* < 0.01 indicates that the difference is extremely significant, and 0.01 < *P* < 0.05 indicates that the difference is significant.

## Results

### *RCI2* Sequence Analysis

On the basis of the known nucleic acid sequences of *MsRCI2A* (JQ665271), *MsRCI2B* (Medtr7g111450), and *MsRCI2C* (Medtr7g111350), the *MsRCI2* genes from *M. sativa* were cloned. Afterward, the 3 *MsRCI2*s and all of the queried Arabidopsis and alfalfa *RCI2* gene members were subjected to sequence alignment to identify conserved regions/sequences (Supplementary Figure 1). The members of the *RCI2* genes were highly homologous, and all had PMP3 protein characteristics. Similar to other PMP3 proteins, the proteins encoded by *MsRCI2* were predicted to have two transmembrane domains according to the probability hypothesis density (PHD) algorithm. A phylogenetic tree based on the amino acid sequences was constructed via the neighbor-joining method (Supplementary Figure 2). The results revealed that *MsRCI2A*, *MsRCI2B*, and *MsRCI2C* clustered into one conserved branch and were homologous to *AtRCI2E*. In addition, *MsRCI2A*/*MtRCI2A*, *MsRCI2B*/*MtRCI2B*, and *MsRCI2C*/*MtRCI2C* emerged as gene pairs, suggesting that they share high sequence identity.

### Expression of the *MsRCI2A, MsRCI2B*, and *MsRCI2C* Genes Under Abiotic Stress

**FIGURE 1:**
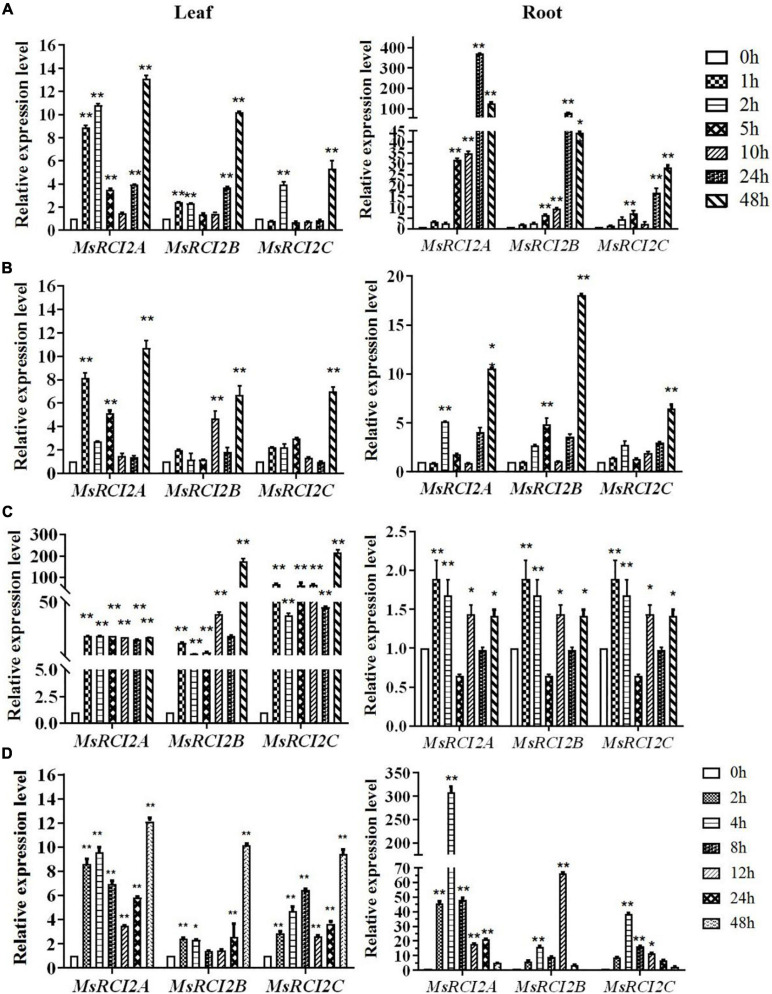
Relative expression of the *MsRCI2* genes under abiotic treatment: **(A)** 100 mmol.L^–1^ NaHCO_3_ (pH 8.5) treatment, **(B)** 200 mmol.L^–1^ NaHCO_3_ (pH 8.5) treatment, **(C)** 200 mmol.L^–1^ NaCl treatment, and **(D)** 100 μmol.L^–1^ ABA induction. The figure on the left shows the expression changes in genes in the leaves; the figure on the right shows those in the roots. The values are the means ± SDs of three replicates (**P < 0.01; *P < 0.05).

As shown in Figure 1, the *MsRCI2A*, *MsRCI2B*, and *MsRCI2C* genes exhibited different expression patterns under alkali and saline stresses, and the same genes were differentially expressed in the roots and leaves. Under 100 mmol.L^–1^ NaHCO_3_ (Figure 1A), the expression of the *MsRCI2* genes in the leaves began to be significantly upregulated early (1 or 2 h) and downregulated at 5–24 h, after which the expression increased at 48 h; under 200 mmol.L^–1^ NaHCO_3_, the expression of the *MsRCI2* genes in the leaves was similar to that under the 100 mmol.L^–1^ NaHCO_3_ treatment. In the roots, under 100 mmol.L^–1^ NaHCO_3_, the expression changes of the *MsRCI2* genes were more significant than those in the leaves; moreover, the expression levels of these genes continuously increased, and they all peaked at 48 h. The expression level of the *MsRCI2A* gene in the roots was obviously significantly greater than those of the *MsRCI2B* and *MsRCI2C* genes. After 200 mmol.L^–1^ NaHCO_3_ (Figure 1B) treatment, the expression of the *MsRCI2* genes was upregulated at 2 h and downregulated at 5 h, and their expression reached the significantly greatest level at 48 h. However, under salt stress (Figure 1C), the expression of the *MsRCI2* genes was most different under alkali stress. After alkali stress, the *MsRCI2* gene had a greater expression level in the roots than in the leaves, while under salt stress, the expression was significantly greater in the leaves than in the roots. Compared with those of *MsRCI2A* and *MsRCI2B*, the expression level of *MsRCI2C* was more significantly upregulated at the early time point (1 h). The expression of all *MsRCI2* genes was upregulated at 1 h after salt treatment and peaked at 48 h in the leaves. Taken together, the above results indicated that the *MsRCI2A*, *MsRCI2B*, and *MsRCI2C* genes respond to alkali and salt stress to different extents.

Under 100 μmol.L^–1^ ABA treatment, the overall changes in the expression levels of the *MsRCI2* genes showed a similar trend to those shown in response to the 100 mmol.L^–1^ NaHCO_3_ treatment (Figure 1D). However, the expression of the *MsRCI2* genes rapidly increased in the roots after treatment but then gradually decreased, reaching a minimum at 48 h. The upregulated expression level of *MsRCI2A* was extremely significant, and the expression levels of these three genes changed more significantly in the roots than in the leaves.

### Generation of Transgenic Alfalfa Plants Overexpressing *MsRCI2A*, *MsRCI2B*, or *MsRCI2C*

Transgenic alfalfa plants overexpressing *MsRCI2A*, *MsRCI2B*, or *MsRCI2C* were generated via the Agrobacterium-mediated cotyledonary node transformation method. After glufosinate selection, 40 independent resistant lines were obtained. The *bar* gene in these plants was detected via PCR, and the results showed that 23 lines were PCR positive. Semiquantitative RT-PCR and quantitative real-time PCR were then conducted to evaluate the mRNA expression levels of the *MsRCI2*s in the PCR-positive plants (Supplementary Figure 3). Transgenic alfalfa plants overexpressing *MsRCI2A*, *MsRCI2B*, or *MsRCI2C* were obtained and named A12 and A22 (plants overexpressing *MsRCI2A*), B13 and B19 (plants overexpressing *MsRCI2B*), and C2 and C10 (plants overexpressing *MsRCI2C*). As shown in Supplementary Figures 3C,D, all of the transcript levels of *MsRCI2*s in the six different types of transgenic plants were significantly greater than those in WT alfalfa plants. Therefore, A12, A22, B13, B19, C2, and C10 were chosen for further phenotypic analysis.

### Changes in the Phenotypic Indexes of Transgenic *MsRCI2* Genes in Alfalfa Under Alkali and Salt Stress

**FIGURE 2:**
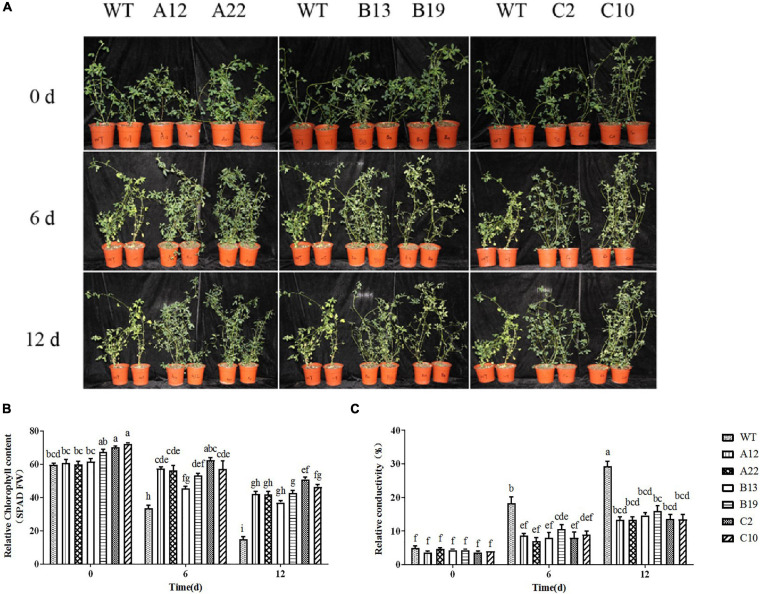
Phenotype **(A)**, Chl content **(B)**, and relative conductivity **(C)** analysis of *MsRCI2* transgenic alfalfa plants under alkali stress. WT stands for wild-type; A12 and A22, B13 and B19, and C2 and C10 were transformed with the *MsRCI2A*, *MsRCI2B*, *MsRCI2C* genes, respectively. The values are the means ± SDs of three replicates (Duncan test: *P* < 0.05).

The phenotypic changes of alfalfa overexpressing *MsRCI2A*, *MsRCI2B*, or *MsRCI2C* under alkali and salt stress are shown in Figure 2 and Supplementary Figure 4. There were no significant differences in the phenotypes, chlorophyll content or relative electrolyte leakage between the A12, A22, B13, B19, C2, and C10 plants and the WT plants when untreated. This meant that overexpression of the *MsRCI2* genes did not alter the growth of alfalfa. After 12 d of treatment with 100 mmol.L^–1^ NaHCO_3_ (pH 8.5) or 200 mmol.L^–1^ NaCl, the overall growth of the WT plants was inhibited, and the plants were nearly dead. The chlorophyll content in the WT plants obviously decreased by 48.7 and 75% after alkali stress and high-salt stress, respectively. The chlorophyll content in the transgenic plants slightly decreased after salt stress (*P* < 0.05) but did not change after alkali stress (Figure 2B and Supplementary Figure 3B). After stress treatment, the relative electrolyte leakage of the WT and transgenic plants increased (Figure 2C and Supplementary Figure 3C), while the rate of increase of the WT plants was significantly greater than that of each transgenic plant, especially under salt stress. When the above results were combined, overexpressing the *MsRCI2* genes could cause alfalfa to be insensitive to both salt and alkali stress, but the chlorophyll content and relative conductivity did not change significantly after stress treatment.

**FIGURE 3:**
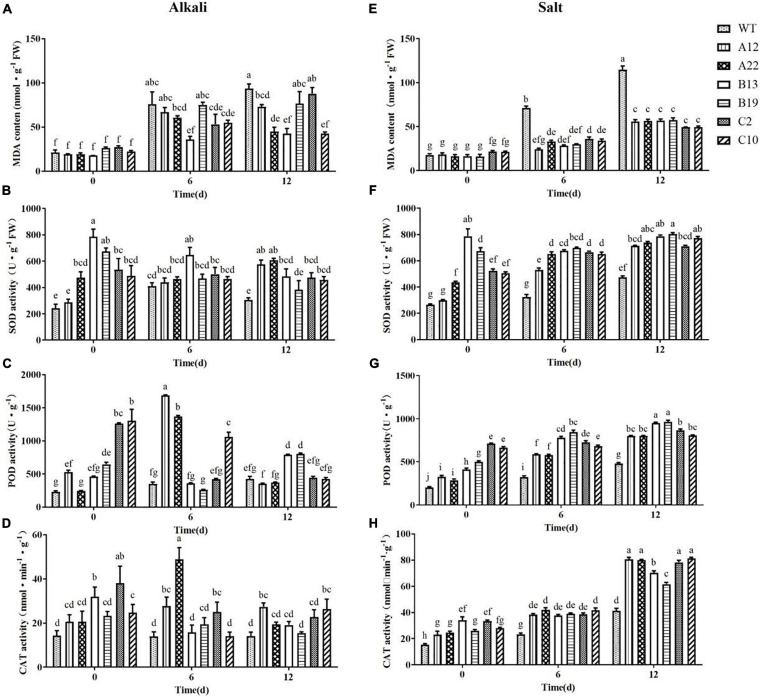
MDA content, SOD activity, POD activity, and CAT activity in WT and transgenic alfalfa plants before and after alkali and salt treatment. Comparisons of the MDA content **(A,E)**, SOD activity **(B,F)**, POD activity **(C,G)**, and CAT activity **(D,H)** of the WT plants and transgenic alfalfa plants before and after 6 and 12 d of alkali and salt treatments. The values are the means ± SDs of three replicates (Duncan test: *P* < 0.05).

The changes in the MDA content under salt and alkali stress were more complex than the changes in the chlorophyll content and electrical conductivity (Figures 3A,E). With respect to WT plants, after 12 days of stress, the MDA content in the WT plants under salt stress was greater than that under alkali stress; that is, plant tissue damage was more severe under salt stress than under alkali stress. For the transgenic plants, after 12 days of salt stress, although the MDA content increased compared with that in the untreated plants, the contents were all significantly lower than those of the WT plants. However, after 12 days of alkali stress, the MDA content in the transgenic plants was not significantly different from that in the WT plants; only in A22, B13, and C10 were the MDA contents significantly lower than that in the WT plants. Similarly, the relative conductivity of the B13 genotype was also obviously lower than those of the others (Figure 2C). Notably, the expression level of the *MsRCI2* gene in the A22 and B13 genotypes was greater than that in the A12 and B19 genotypes (Supplementary Figure 3D).

### Changes in the Antioxidant Capacity of *MsRCI2* Transgenic Alfalfa Under Alkali and Salt Stress

Under normal conditions, there were significant differences in the SOD, POD, and CAT activities between several transgenic plants and WT plants. After alkali treatment, the SOD enzyme activity in the transgenic plants changed only slightly: the activity in A12 and A22 increased, that in B13 and B19 decreased, and that in C2 and C10 did not change. After 12 days of alkali stress, only A12 and A22 presented greater SOD activity than the WT (*P* < 0.05) (Figure 3B).

As shown in Figure 3C, the POD activity in A12, B13, B19, C2, and C10 was significantly greater than that in the WT (*P* < 0.01) without treatment, especially in C2 and C10. Interestingly, after 6 days of alkali treatment, the POD activity in the WT plants B13, B19, and C10 did not obviously change; however, that in A12 and A22 greatly increased, and that in C2 sharply decreased (*P* < 0.01). After 12 days of alkali treatment, the POD activity in A12, A22, and C10 significantly decreased, and only in B13 and B19 was the POD activity significantly greater than that in the other genotypes (*P* < 0.01). The above results indicated that, compared with that in the other plants, the POD activity in the B13 and B19 plants lasted longer under alkali stress, and compared with that in A12 and A22, it responded more quickly. There were significant differences in the CAT activity of B13 and C2 compared with the other genotypes under normal conditions. After alkali treatment, the CAT activity in B13 and C2 decreased to the same level as that in the WT on the 12th day (Figure 3D).

For salinity stress, as shown in Figures 3F–H, the activities of SOD, POD, and CAT in each transgenic plant increased (*P* < 0.01), while the antioxidant capacity of the WT slightly increased only on the 12th day (*P* < 0.05). From the changes in SOD, POD, and CAT enzyme activities, it could be seen that, under salt stress, each transgenic plant presented greater amounts of SOD activity than the WT did and had a stronger ability to remove reactive oxygen species (ROS). In addition, during the salt stress response, the three MsRCI2A, MsRCI2B, and MsRCI2C proteins had similar functions.

### Changes in Osmotic Adjustment Substances in *MsRCI2* Transgenic Alfalfa Plants Under Alkali and Salt Stress

**FIGURE 4:**
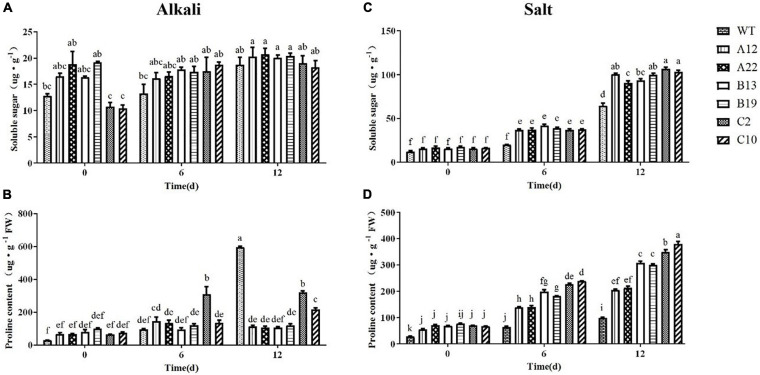
Contents of soluble sugars and Pro in WT and transgenic alfalfa plants before and after alkali and salt treatments. Comparisons of soluble sugar contents **(A,C)** and Pro contents **(B,D)** of WT and transgenic alfalfa plants before and after 6- and 12-days alkali and salt treatments. The values are the means ± SDs of three replicates (Duncan test: *P* < 0.05).

As shown in Figure 4, after salt and alkali stress, the changes in soluble sugar and proline contents were quite different. Under alkali stress, the soluble sugar content in all of the plants did not significantly increase. However, the soluble sugar content in all of the plants obviously increased, and that in the transgenic plants increased significantly under salt stress. Under normal conditions, compared with the WT plants, the transgenic plants had a greater proline content, but there were differences under alkali and salt stress. Under alkali stress, the increase in the WT plants was significantly greater than that in the transgenic plants, but the opposite was true under salt stress. Notably, the increase in C2 and C10 was greater than that in the other transgenic plants under alkali and salt stress.

### Changes in the Expression of Stress-Responsive Genes in Transgenic Alfalfa Plants Under Alkali Stress

**FIGURE 5:**
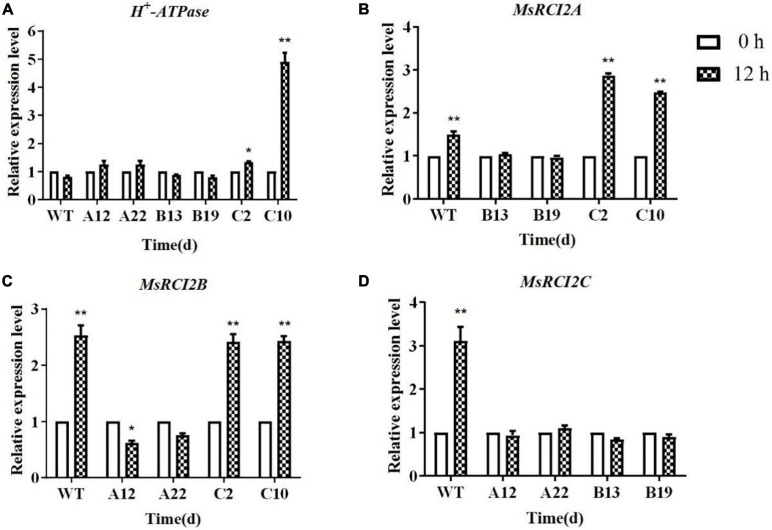
Relative expression of stress-responsive *H*^+^-*ATPase* **(A)**, *MsRCI2A* **(B)**, *MsRCI2B* **(C)**, and *MsRCI2C* **(D)** genes in WT and transgenic alfalfa plants under alkali stress. The values are the means ± SDs of three replicates (**P < 0.01; *P < 0.05).

To further explore the changes in the expression of alkali stress-related genes in the transgenic plants, the expression levels of *H^+^-ATPase* genes were analyzed (Figure 5A). After treatment with 100 mmol.L^–1^ NaHCO_3_ (pH 8.5) for 12 h, the expression of the *H^+^-ATPase* genes in the WT and transgenic plants was not different, except in the C10 genotype.

*MsRCI2A*, *MsRCI2B*, and *MsRCI2C* are highly homologous, and all respond to stress with different levels of expression, which manifests as differences in physiological indicators between different transgenic plants, especially under alkali stress. Therefore, the expression of the *RCI2* genes in each transgenic plant under alkali stress was analyzed (Figures 5B–D). After 12 h of alkali treatment, the expression of the *MsRCI2A* gene in the cells of B13 and B19 was slightly suppressed, while the expression of the *MsRCI2A* gene was upregulated in the cells of C2 and C10 (*P* < 0.01) (Figure 5B). For *MsRCI2B*, the expression in C2 and C10 was not different from that in the WT, while in A12 and A22, the expression was downregulated (Figure 5C). Moreover, the expression of the *MsRCI2C* gene in the cells of A12, A22, B13, and B19 was significantly suppressed (Figure 5D). This meant that there is a potential regulatory relationship among *MsRCI2A*, *MsRCI2B*, and *MsRCI2C* in the alkali stress response and that their functions are non-redundant.

## Discussion

**FIGURE 6:**
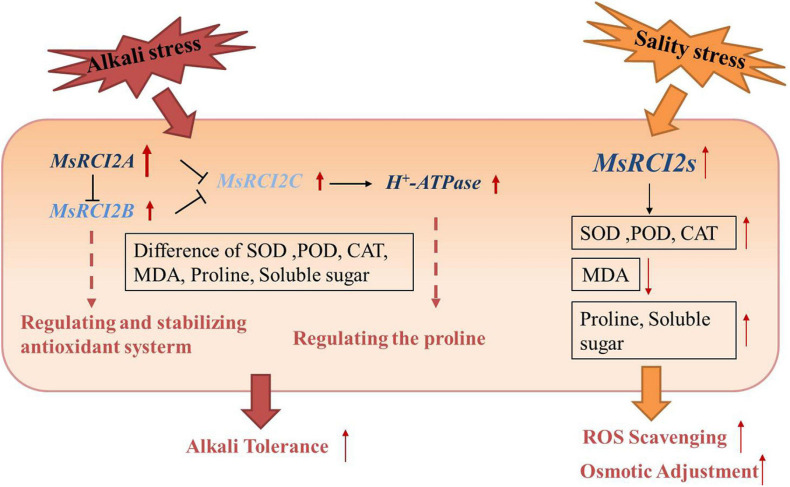
Functional model of the *MsRCI2* response to alkali and salinity stress. The main functions of *MsRCI2s* are as follows: (1) ROS scavenging and osmotic adjustment in response to salinity stress; (2) differences in *MsRCI2* gene expression and regulatory relationships with *H^+^-ATPase* or *MsRCI2*s; and (3) overexpression of *MsRCI2A/B*/*C* genes enhanced alkali and salt tolerance.

Several studies have shown that small plasma membrane-localized proteins encoded by members of the *PMP3/RCI2* gene family in plants can stabilize the ion balance in cells and avoid excessive Na^+^ absorption by regulating ion transporters or other membrane proteins (Capel et al., 1997; Mitsuya et al., 2005, 2006). *RCI2* genes are involved in a variety of abiotic stresses. For example, in transformed tobacco plants, the expression of *AltMP1* and *AltMP2* transgenes increases the stability of the cell membrane, maintains the ion balance in the cells and induces the expression of stress-related genes to improve tolerance to drought, low temperature, salt, heat shock, osmotic stress and H_2_O_2_ stress (Ben et al., 2017, 2018). Nearly all studies on the overexpression of the *PMP3* gene have shown that transgenic plants present enhanced resistance to abiotic stress (Rocha, 2016; Kim et al., 2021, 2020). In this study, the *MsRCI2A*, *MsRCI2B*, and *MsRCI2C* genes were cloned from alfalfa and further analyzed. Consistent with the findings of previous studies, *MsRCI2A*, *MsRCI2B*, and *MsRCI2C* overexpression improved the salt and alkali tolerance of alfalfa; however, in response to alkali stress, the three proteins encoded by *MsRCI2s* exhibited functional differences (Figure 6).

### Responses of the *MsRCI2A, MsRCI2B*, and *MsRCI2C* Genes to Abiotic Stress

Plants respond to abiotic stress by continuously altering their level of ABA, and these alterations lead to stomatal closure; induce the expression of stress-related genes; increase the synthesis of metabolites, carbohydrates, and LEA proteins; and increase the activity of antioxidant enzymes (Kim et al., 2004; Vysotskii et al., 2013; Wang et al., 2013). In this study, the expression of *MsRCI2A*, *MsRCI2B*, and *MsRCI2C* in alfalfa was significantly upregulated in both the leaves and roots in response to ABA, and the gene expression changes in the roots were greater than those in the leaves (Figure 1D). The three proteins accumulate rapidly under ABA induction, indicating that *MsRCI2A*, *MsRCI2B*, and *MsRCI2C* participate in the early response mechanism of abiotic stress, especially in the roots, and that they are all regulated by ABA-dependent pathways.

To compare the differences in the response to salt and alkali stresses, tests of the effects of three different concentrations of salt and alkali stress were designed. The concentration of 100 mmol.L^–1^ NaHCO_3_ was considered to be an alkali stress with a low ion concentration, 200 mmol.L^–1^ NaCl was the high ion-concentration salt stress, and 200 mmol.L^–1^ NaHCO_3_ was the salt-alkali dual stressor. The *MsRCI2A*, *MsRCI2B*, and *MsRCI2C* genes exhibited upregulated expression patterns in both the roots and leaves of plants under 100 and 200 mmol.L^–1^ NaHCO_3_ and 200 mmol.L^–1^ NaCl stress treatments. However, the range of variation was different. This indicates that even though *MsRCI2A*, *MsRCI2B*, and *MsRCI2C* are highly homologous, they have different responses to salt and alkali stress and might therefore have different functions. Notably, the expression patterns of *MsRCI2A*, *MsRCI2B*, and *MsRCI2C* in the leaves under alkali stress were similar to those under ABA treatment, and the gene expression levels in the roots were also significantly greater than those in the leaves. Perhaps there was a more direct regulation between alkali stress and ABA. It has also been reported in other plant species that the expression of genes homologous to *PMP3* can be upregulated or downregulated in response to different abiotic stress stimuli and that the expression of *RCI* genes under normal conditions also shows tissue and organ specificity (Wang et al., 2013; Rocha, 2016).

### Different Functions of the *MsRCI2* Genes Under Salt and Alkali Stress

Many studies have shown that adaptive strategies in response to salt and alkali stresses are quite different (Yang et al., 2009; Wang et al., 2012). Differences in gene expression have indicated this. However, in this study, from the changes in various indicators of the WT plants under stress, we could also conclude that high-salinity stress (200 mmol.L^–1^ NaCl) had a more significant impact on alfalfa than weak alkali stress (100 mmol.L^–1^ NaHCO_3_, pH 8.5). After salt stress, the changes in the chlorophyll content, relative electrolyte leakage and MDA were more obvious under salt stress than under alkali stress (Figures 2B,C, 3A,E and Supplementary Figures 4B,C), and the proline content increased significantly under alkali stress (Figure 4). With respect to ion stress, alfalfa was not sensitive to 100 mmol.L^–1^ Na^+^ and still had the ability to osmotically adjust, however, alfalfa was more sensitive to 200 mmol.L^–1^ Na^+^, under which its osmotic adjustment ability decreased, and the proline change was not significant. Therefore, the damage of low concentrations of Na^+^ ions to alfalfa was much lower under 100 mmol.L^–1^ Na^+^ than under 200 mmol.L^–1^ Na^+^.

In this study, *MsRCI2A*, *MsRCI2B*, and *MsRCI2C* overexpression improved the salt and alkali tolerance of alfalfa. The data showed that, under normal conditions, certain physiological indicators of transgenic plants were different from those of the WT plants, which was conducive to the stress tolerance of the plants. For example, the SOD, POD, and CAT enzyme activities and proline content in several transgenic plants were significantly greater than those in the WT plants. Additional studies have also concluded that Pro accumulation under salt stress is correlated with stress tolerance and that proline concentrations have been shown to be generally greater in salt-tolerant plants than in salt-sensitive plants (Hayat et al., 2012; Verma et al., 2020). In addition to being an osmolyte for osmotic adjustment, proline also scavenges free radicals and buffers the cellular redox potential under stress conditions, such as the production of ROS (Hayat et al., 2012).

There were also differences in the antioxidant enzyme activity and proline content between salt and alkali stress. Under salt stress, the SOD, POD, and CAT activities and proline content of all the transgenic plants increased (Figures 3F–H, 4D). However, under alkali stress, there were different changes among different transgenic plants, and these differences were directly related to the three *MsRCI2* genes. With respect to the antioxidant enzyme activity, in A12/A22, the POD activity first increased sharply but then significantly decreased, while in B13/B19, it increased slightly. After alkaline treatment for 12 days, the POD activity in only B13/B19 was significantly greater than that in the WT plants (Figure 3C). The Pro content increased only in C2/C10 but was lower than that in the WT plants, while the content in the other transgenic plants did not obviously change. On the basis of the above results, MsRCI2A, MsRCI2B, and MsRCI2C had the same function in response to salt stress. However, MsRCI2A was more prominent in the regulation of antioxidant enzymes, and MsRCI2B also played a certain role in stabilizing the activity of antioxidant enzymes. Only MsRCI2C was involved in regulating the proline content in response to alkali stress.

Additional studies have focused on the function of the *RCI2* protein in regulating ion balance under salt stress (Mitsuya et al., 2006; Ben et al., 2017, 2018). Few studies have paid attention to the role of alkali stress as well as its role in osmotic regulation and removal of ROS. In this study, it was shown that the protein encoded by the MsRCI2 genes could regulate the contents of proline and soluble sugar under salt or alkali stress, even though there were differences between the effects of the three *MsRCI2* genes. Due to their small size, PMP3/RCI2s are thought to not be ion transporters (Nylander et al., 2001). The possibility that RCI2s may exert their effects through interactions with other proteins is further highlighted by the physical interactions detected between yeast Pmp3p and several permeases, including the amino acid permeases Gap1 and Agp1, the maltose permease Mal31, the S-methylmethionine permease Mmp1, and Ydr307w (an interactor of Mep2 ammonia permease) (Miller et al., 2005; Van Zeebroeck et al., 2011). The transport of these enzymes in the cell changes the osmotic potential of the cell. However, how *MsRCI2* genes directly or indirectly alter the proline content, thereby affecting the activity of antioxidant enzymes, remains to be further studied.

### Interactions Between RCI2 Genes and the Regulation of H^+^-ATPase Enzymes

Plant RCI2s are thought to function like Pmp3p in regulating ion homeostasis, and RCI2s may affect ion transporters by regulating ion transport proteins or other membrane proteins (Nylander et al., 2001). H^+^-ATPases act as vacuolar membrane proton pumps, and multiple studies have shown that H^+^-ATPases can maintain intracellular pH levels under alkali stress (Wang and Zou, 2000; Sun et al., 2014). Liu showed that the activity of H^+^-ATPases in all studied Arabidopsis lines decreased under salt stress. However, among WT plants, *rci2a* mutants, and 35S:*MpRCI*-rci2a transgenic plants, the H^+^-ATPase activity in the *rci2a* mutants was the most significantly reduced, which indicates that the *AtRCI2A* and *MpRCI* genes affect the activity of H^+^-ATPases (Liu et al., 2012). To further study the mechanism underlying the response of the *MsRCI2A*, *MsRCI2B*, and *MsRCI2C* genes to alkali stress, the expression of *H^+^-ATPase* genes in each genotype after 12 h of alkaline treatment was analyzed via qPCR (Figure 5A). Interestingly, only in transgenic genotypes C2 and C10 was the expression of *H^+^-ATPase* upregulated; in the other transgenic genotypes, there were no differences compared with the expression in the WT plants. In addition, the expression level of *H^+^-ATPase* was greater in C10 than in C2, and the *MsRCI2C* gene expression level was lower in C10 than in C2 (Supplementary Figure 3D). This indicates that there was a regulatory relationship between *H^+^-ATPase* and *MsRCI2C* and that the expression level of *H^+^-ATPase* was related to *MsRCI2C*, which maintained the intracellular pH.

Most researchers believe that, due to their small size, RCI2s cannot be ion transporters alone. However, this does not prevent two RCI2 oligomers from potentially jointly forming a transporter or interacting with other membrane proteins. Kim et al. (2019) found that NaCl-induced *CsRCI2E* and *CsRCI2F* interact with aquaporin CsPIP2; 1 to reduce water transport. In this study, we found that overexpression of *MsRCI2A* or *MsRCI2B* downregulated the expression of the *MsRCI2C* and *MsRCI2B* genes, while overexpression of *MsRCI2C* slightly upregulated the expression of *MsRCI2A* but did not affect the expression of *MsRCI2B* (Figures 5B–D). This indicates that the expression of the *MsRCI2A*, *MsRCI2B*, and *MsRCI2C* genes is mutually regulated. However, it is still uncertain whether physical interactions occur among MsRCI2 proteins. Future work should address the mechanisms by which RCI2s affect membrane properties, participate in ion homeostasis, regulate pH and interact with other proteins. The identification of protein interaction partners of RCI2s is critical to future studies.

## Conclusion

The expression of *MsRCI2A*, *MsRCI2B*, and *MsRCI2C* was induced by salt, alkali, and ABA treatment, but there were differences between the expression of *MsRCI2* genes under different treatments. Overexpression of *MsRCI2A*, *MsRCI2B*, and *MsRCI2C* can improve the alkali and salt tolerance of alfalfa. Overexpression of *MsRCI2*s in alfalfa yielded the same physiological response to salt stress. However, in response to alkali stress, the three studied proteins encoded by *MsRCI2s* exhibited functional differences, which were determined not only by their different modes of transcription but also by the differences in their regulatory relationship with H^+^-ATPase or other MsRCI2s.

## Data Availability Statement

The original contributions presented in the study are included in the article/Supplementary Material, further inquiries can be directed to the corresponding author/s.

## Author Contributions

HC and CL designed and conducted the study and drafted the manuscript. TS, LZ, CC, HX, and LD performed the experiments. All authors contributed to the acquisition of data, interpretation of results and critical discussion and approved the final version of the manuscript.

## Conflict of Interest

The authors declare that the research was conducted in the absence of any commercial or financial relationships that could be construed as a potential conflict of interest.

## Publisher’s Note

All claims expressed in this article are solely those of the authors and do not necessarily represent those of their affiliated organizations, or those of the publisher, the editors and the reviewers. Any product that may be evaluated in this article, or claim that may be made by its manufacturer, is not guaranteed or endorsed by the publisher.

## Footnotes

1
https://phytozome.jgi.doe.gov

2
https://www.megasoftware.net/

## Abbreviations

ABA: abscisic acid
CAT: catalase
MDA: malondialdehyde
PMP3: plasma membrane protein 3
PHD: probability hypothesis density
POD: peroxidase
Pro: proline
RCI2: Rare cold-inducible 2
SA: salicylic acid
SOD: superoxide dismutase
WT: wild type.

## References

[B1] Ben R. W. Ben S. R. Meynard D. Verdeil J. L. Azaza J. Zouari N. (2017). Ectopic expression of Aeluropus littoralis plasma membrane protein gene AlTMP1 confers abiotic stress tolerance in transgenic tobacco by improving water status and cation homeostasis. *Int. J. Mol. Sci.* 18:692. 10.3390/ijms18040692 28338609 PMC5412278

[B2] Ben R. W. Ben S. R. Meynard D. Zouari N. Mahjoub A. Fki L. (2018). Overexpression of AlTMP2 gene from the halophyte grass Aeluropus littoralis in transgenic tobacco enhances tolerance to different abiotic stresses by improving membrane stability and deregulating some stress-related genes. *Protoplasma* 255 1161–1177. 10.1007/s00709-018-1223-3 29450758

[B3] Capel J. Jarillo J. A. Salinas J. Martinez-Zapater J. M. (1997). Two homologous low-temperature-inducible genes from Arabidopsis encode highly hydrophobic proteins. *Plant Physiol.* 115 569–576. 10.1104/pp.115.2.569 9342870 PMC158516

[B4] Dong X. Im S. B. Lim Y. P. Nou I. S. Hur Y. (2014). Comparative transcriptome profiling of freezing stress responsiveness in two contrasting Chinese cabbage genotypes, Chiifu and Kenshin. *Genes Genomics* 36 215–227. 10.1007/s13258-013-0160-y

[B5] Fu J. Zhang D. F. Liu Y. H. Ying S. Shi Y. S. Song Y. C. (2012). Isolation and characterization of maize PMP3 genes involved in salt stress tolerance. *PLoS One* 7:e31101. 10.1371/journal.pone.0031101 22348040 PMC3278423

[B6] Hayat S. Hayat Q. Alyemeni M. N. Wani A. S. Pichtel J. Ahmad A. (2012). Role of proline under changing environments: a review. *Plant Signal. Behav.* 7 1456–1466. 10.4161/psb.21949 22951402 PMC3548871

[B7] Huihui Z. Xin L. Yupeng G. Mabo L. Yue W. Meijun A. (2020). Physiological and proteomic responses of reactive oxygen species metabolism and antioxidant machinery in mulberry (*Morus alba* L.) seedling leaves to NaCl and NaHCO3 stress. *Ecotoxicol. Environ. Saf.* 193:110259. 10.1016/j.ecoenv.2020.110259 32097787

[B8] Kim H. S. Lee J. E. Jang H. Y. Jin K. K. Ahn S. J. (2016). CsRCI2A and CsRCI2E genes show opposite salt sensitivity reaction due to membrane potential control. *Acta Physiol. Plant.* 38:50. 10.1007/s11738-016-2072-3

[B9] Kim H. S. Park W. Lee H. S. Shin J. H. Ahn S. J. (2021). Subcellular Journey of Rare Cold Inducible 2 Protein in Plant Under Stressful Condition. *Front. Plant Sci.* 11:610251. 10.3389/fpls.2020.610251 33510753 PMC7835403

[B10] Kim H. S. Park W. Lim H. G. Eom S. Lee J. H. Carlson J. E. (2019). NaCl-induced CsRCI2E and CsRCI2F interact with aquaporin CsPIP2; 1 to reduce water transport in *Camelina sativa* L. *Biochem. Biophys. Res. Commun.* 513 213–218. 10.1016/j.bbrc.2019.03.208 30954220

[B11] Kim J. B. Kang J. Y. Kim S. Y. (2004). Over-expression of a transcription factor regulating ABA-responsive gene expression confers multiple stress tolerance. *Plant Biotechnol. J.* 2 459–466. 10.1111/j.1467-7652.2004.00090.x 17168892

[B12] Kim S. H. Kim J. Y. Kim S. J. An K. S. An G. Kim S. R. (2007). Isolation of cold stress-responsive genes in the reproductive organs, and characterization of the OsLti6b gene from rice (*Oryza sativa* L.). *Plant Cell Rep.* 26 1097–1110. 10.1007/s00299-006-0297-0 17219102

[B13] Kim Y. O. Lim H. G. Kim H. S. Ahn S. J. (2020). Overexpression of CsRCI2H enhances salt tolerance in *Camelina sativa* (L.). *Plant Biotechnol. Rep.* 14 439–449. 10.1007/s11816-020-00622-9

[B14] Liu B. Feng D. Zhang B. Mu P. Zhang Y. He Y. (2012). Musaparadisica RCI complements AtRCI and confers Na+ tolerance and K+ sensitivity in Arabidopsis. *Plant Sci.* 184 102–111. 10.1016/j.plantsci.2011.12.004 22284714

[B15] Long R. Zhang F. Li Z. Li M. Cong L. Kang J. (2015). Isolation and functional characterization of salt-stress induced RCI2-like genes from *Medicago sativa* and *Medicago truncatula*. *J. Plant Res.* 128 697–707. 10.1007/s10265-015-0715-x 25801273

[B16] Miller J. P. Lo R. S. Ben-Hur A. Desmarais C. Stagljar I. Noble W. S. (2005). Large-scale identification of yeast integral membrane protein interactions. *Proc. Natl. Acad. Sci. U. S. A.* 102 12123–12128. 10.1073/pnas.0505482102 16093310 PMC1189342

[B17] Mitsuya S. Taniguchi M. Miyake H. Takabe T. (2005). Disruption of RCI2A leads to over-accumulation of Na+ and increased salt sensitivity in Arabidopsis thaliana plants. *Planta* 222 1001–1009. 10.1007/s00425-005-0043-9 16034593

[B18] Mitsuya S. Taniguchi M. Miyake H. Takabe T. (2006). Overexpression of RCI2A decreases Na+ uptake and mitigates salinity-induced damages in Arabidopsis thaliana plants. *Physiol. Plant* 128 95–102. 10.1111/j.1399-3054.2006.00714.x

[B19] Nylander M. Heino P. Helenius E. Palva E. T. Ronne H. Welin B. V. (2001). The low-temperature-and salt-induced RCI2A gene of Arabidopsis complements the sodium sensitivity caused by a deletion of the homologous yeast gene SNA1. *Plant Mol. Biol.* 45 341–352. 11292079 10.1023/a:1006451914231

[B20] Permingeat H. R. Romagnoli M. V. Sesma J. I. Vallejos R. H. (1998). A simple method for isolating DNA of high yield and quality from cotton (*Gossypium hirsutum* L.) leaves. *Plant Mol. Biol. Rep.* 16:89. 10.1023/A:1007466522028

[B21] Rocha P. S. (2016). Plant abiotic stress-related RCI2/PMP3s: multigenes for multiple roles. *Planta* 243 1–12. 10.1007/s00425-015-2386-1 26306604

[B22] Song T. Sun N. Dong L. Cai H. (2021). Enhanced alkali tolerance of rhizobia-inoculated alfalfa correlates with altered proteins and metabolic processes as well as decreased oxidative damage. *Plant Physiol. Biochem.* 159 301–311. 10.1016/j.plaphy.2020.12.021 33418189

[B23] Sun M. Jia B. Cui N. Wen Y. Duanmu H. Yu Q. (2016). Functional characterization of a Glycine soja Ca2+ ATPase in salt-alkaline stress responses. *Plant Mol. Biol.* 90 419–434. 10.1007/s11103-015-0426-7 26801329

[B24] Sun M. Sun X. Zhao Y. Zhao C. Duanmu H. Yu Y. (2014). Ectopic expression of GsPPCK3 and SCMRP in *Medicago sativa* enhances plant alkaline stress tolerance and methionine content. *PLoS One* 9:e89578. 10.1371/journal.pone.0089578 24586886 PMC3934933

[B25] Sun N. Song T. Ma Z. Dong L. Zhan L. Xing Y. (2020). Overexpression of MsSiR enhances alkali tolerance in alfalfa (*Medicago sativa* L.) by increasing the glutathione content. *Plant Physiol. Biochem.* 154 538–546. 10.1016/j.plaphy.2020.07.001 32912487

[B26] Van Zeebroeck G. Kimpe M. Vandormael P. Thevelein J. M. (2011). A split-ubiquitin two-hybrid screen for proteins physically interacting with the yeast amino acid transceptor Gap1 and ammonium transceptor Mep2. *PLoS One* 6:e24275. 10.1371/journal.pone.0024275 21912684 PMC3166329

[B27] Verma D. Jalmi S. K. Bhagat P. K. Verma N. Sinha A. K. (2020). A bHLH transcription factor, MYC2, imparts salt intolerance by regulating proline biosynthesis in Arabidopsis. *FEBS J.* 287 2560–2576. 10.1111/febs.15157 31782895

[B28] Vysotskii D. A. de Vries-van Leeuwen I. J. Souer E. Babakov A. V. de Boer A. H. (2013). ABF transcription factors of Thellungiella salsuginea: structure, expression profiles and interaction with 14-3-3 regulatory proteins. *Plant Signal. Behav.* 8:e22672. 10.4161/psb.22672 23221757 PMC3745569

[B29] Wang B. S. Zou Q. (2000). Advances in the study on Plasma Membrane-bound Translocating Proteins and Their Relations with Salt Tolerance in Plants. *Chin. Bull. Bot.* 17 17–26.

[B30] Wang D. Chen Y. Wang Y. Wang Z. (2013). Molecular cloning and expression of two plasma membrane protein 3 (SmPMP3) genes from Salvia miltiorrhiza. *Russ. J. Plant Physiol.* 60 155–164. 10.1134/s1021443712060179

[B31] Wang X. P. Chen W. C. Zhou Y. Han J. Y. Zhao J. Shi D. C. (2012). Comparison of adaptive strategies of alfalfa (*Medicago sativa* L.) to salt and alkali stresses. *AJCS* 6 309–315.

[B32] Yang C. W. Xu H. H. Wang L. L. Liu J. Shi D. C. Wang D. L. (2009). Comparative effects of salt-stress and alkali-stress on the growth, photosynthesis, solute accumulation, and ion balance of barley plants. *Photosynthetica* 47 79–86. 10.1007/s11099-009-0013-8

[B33] Yang H. Q. (2017). Analysis of Nutritional Components of alfalfa. *Feed Pasture Dev.* 11:91.

