# Supplementary material for: Overexpression of MsRCI2A, MsRCI2B, and MsRCI2C in Alfalfa (Medicago sativa L.) Provides Different Extents of Enhanced Alkali and Salt Tolerance Due to Functional Specialization of MsRCI2s
Source: Front Plant Sci. 2021 Aug 19;12:702195. doi: 10.3389/fpls.2021.702195 (PMC8417119; doi:10.3389/fpls.2021.702195)
Supplement: Supplementary file 1 [file Data_Sheet_1.docx]

Table 1 Information of primers used in the study

| Primer name | Primer sequence（5’— 3’） | Purpose |
| --- | --- | --- |
| *MsRCI2A-F* | ATATTGAAAATGGGCACTGCTAC | Gene cloning and qPCR |
| *MsRCI2A-R* | TGGAAGATGATGATATGGATCACT | Gene cloning and qPCR |
| *MsRCI2B-F* | ATGGGCACAGCTACATGCATC | Gene cloning and qPCR |
| *MsRCI2B-R* | TCACTTGGTAATAGCATAGATAGCATAGA | Gene cloning and qPCR |
| *MsRCI2C-F* | ATGGGCACAGCTACCTTCG | Gene cloning and qPCR |
| *MsRCI2C-R* | TCACTTAGTGATAATATAGATAGCATAGAT | Gene cloning and qPCR |
| *H^+^*-*ATPase-F* | ATGGGAGCAGTAATTCTCCC | Gene cloning and qPCR |
| *H^+^*-*ATPase-R* | TTAGATCTTGAAGAGGAGACCACC | Gene cloning and qPCR |
| MDC-*MsRCI2A-F* | CGACTCTAGAGGATCCCCGGGATATTGAAAATGGGCACTGCTAC | Vector construction |
| MDC-*MsRCI2A-R* | GGCGGCCGCTCTAGAACTAGTGGAAGATGATGATATGGATCACT | Vector construction |
| MDC-*MsRCI2B-F* | CGACTCTAGAGGATCCCCGGGATGGGCACAGCTACATGCATC | Vector construction |
| MDC-*MsRCI2B-R* | GGCGGCCGCTCTAGAACTAGTTCACTTGGTAATAGCATAGATAGCATAGA | Vector construction |
| MDC-*MsRCI2C-F* | CGACTCTAGAGGATCCCCGGGATGGGCACAGCTACCTTCG | Vector construction |
| MDC-*MsRCI2C-R* | GGCGGCCGCTCTAGAACTAGTTCACTTAGTGATAATATAGATAGCATAGAT | Vector construction |
| *Bar-F* | ATGAGCCCAGAACGACGC | PCR analysis |
| *Bar-R* | TCAAATCTCGGTGACGGGC | PCR analysis |
| *GAPDH-F* | GGCTGCATCAAGGAGGAAT | RT-PCR reference |
| *GAPDH-R* | TCCAAGCTCAGCCTCATCAAG | RT-PCR reference |

Table 2 Accession number of *RCI2* gene family members in alfalfa and Arabidopsis

| Gene ID | Gene name |
| --- | --- |
| JQ665271 | *MsRCI2A* |
| Medtr7g111380  Medtr7g111450 | *MtRCI2A*  *MtRCI2B* |
| Medtr7g111350  Medtr6g033495  Medtr4g130660  Medtr4g130675  AT3G05880  AT3G05890  AT1G57550  AT2G24040  AT4G30650  AT4G30660  AT4G28088  AT2G38905 | *MtRCI2C*  *MtRCI2D*  *MtRCI2E*  *MtRCI2F*  *AtRCI2A*  *AtRCI2B*  *AtRCI2C*  *AtRCI2D*  *AtRCI2E*  *AtRCI2F*  *AtRCI2G*  *AtRCI2H* |


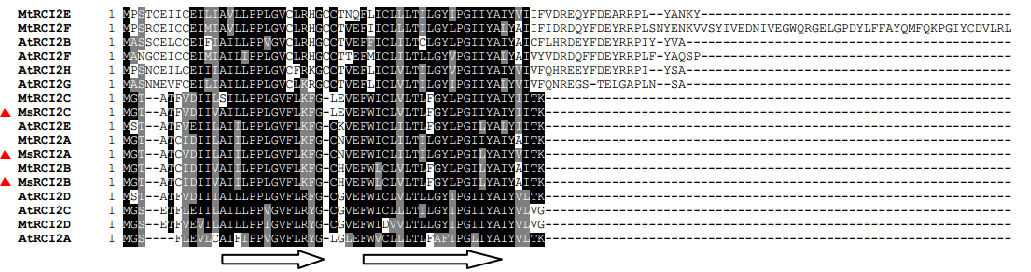


Fig.1 Amino acid sequence alignment of *RCI2* genes in alfalfa and *Arabidopsis.*Identical sequences were marked in black and the arrows indicate two hypothetical transmembrane domains.


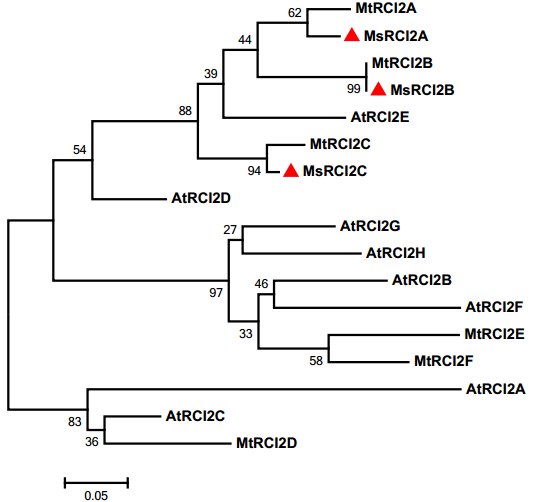


Fig. 2. Phylogenetic tree representing relationships among RCI2 proteins from Alfalfa and *Arabidopsis*.The phylogenetic tree was constructed using MEGA6 based on the Neighbor-Joining (NJ) method;bootstrap was 1000 replicates.The values on the different-length branches are the distance values, which represent the probability of the appearance of drawing 1000 evolutionary trees.


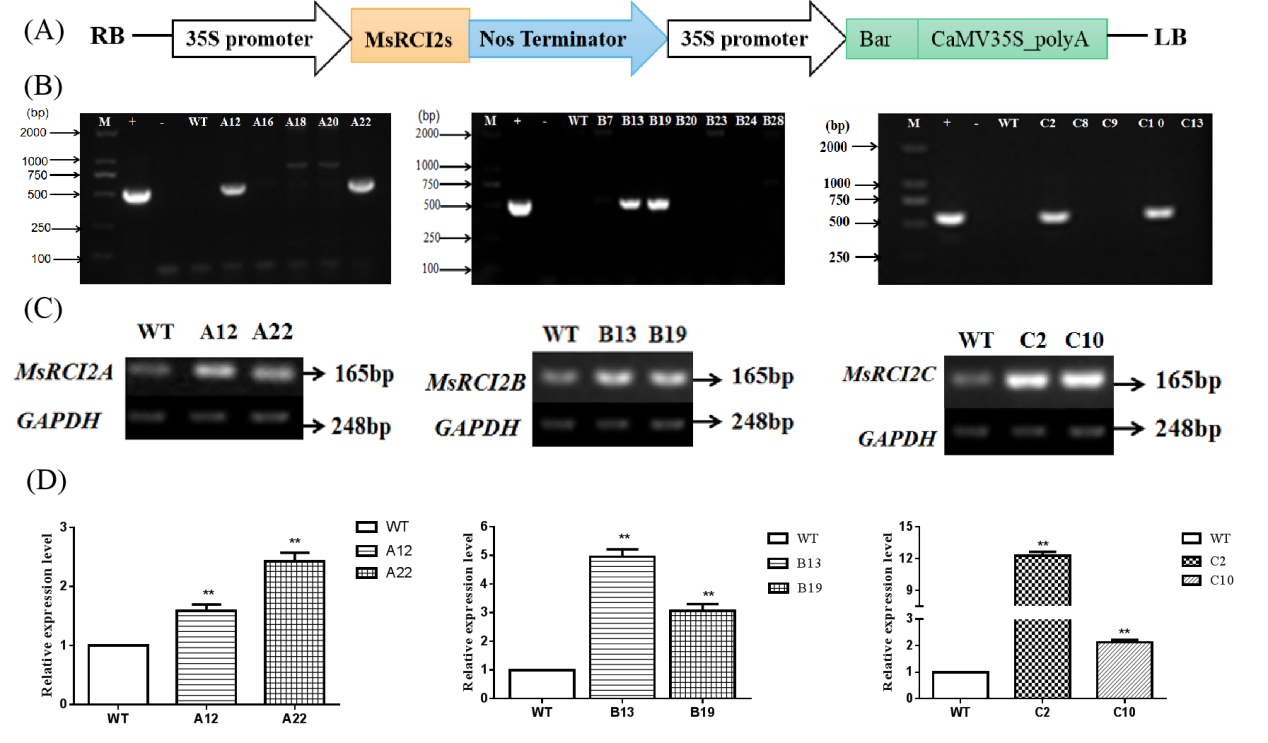


Fig. 3. Generation and molecular tests of *MsRCI2s* transgenic plants. (A) The schematic map of the T-DNA region of the *MsRCI2s* expression vector. LB, left border; RB, right border;(B) PCR identification of transformation with *MsRCI2s*. (C) RT-PCR identification of transformation with *MsRCI2s*. M:Trans2K DNA Marker; +: Positive control; -: ddH_2_O negative control; WT:non-transgenic lines; A12,A16,A18,A20,A22:Transgenic plants with *MSRCI2A* gene. B7, B13, B19, B20, B23, B24, B28:Transgenic plants with *MSRCI2B* gene. C2,C8,C9,C10 and C13: Transgenic plants with *MsRCI2C* gene.(D) Relative expression of *MsRCI2s* genes in WT and *MsRCI2s* overexpression lines A12,A22,B13,B19,C2 and C10.


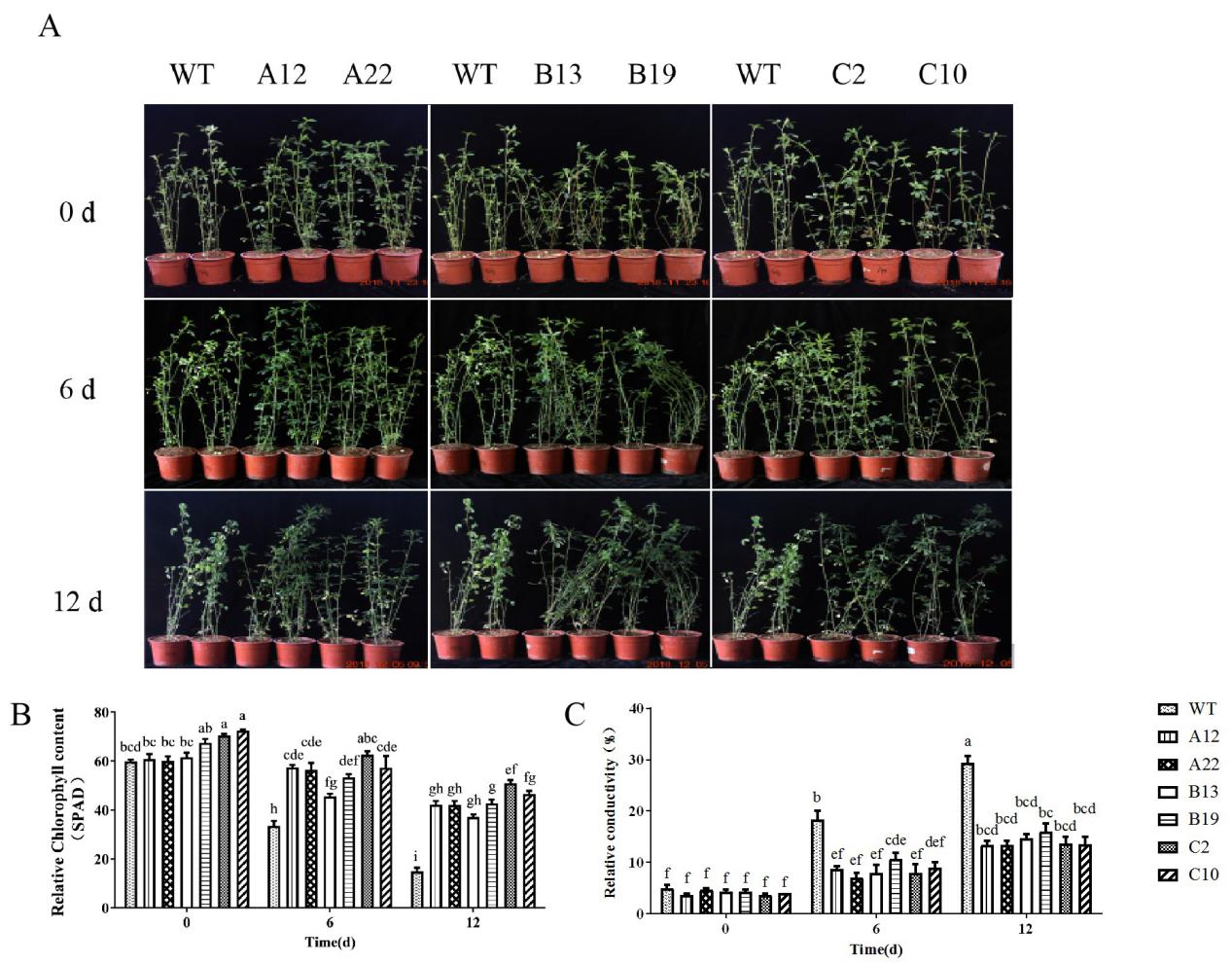


Fig.4. Phenotype(A), chlorophyll content (B) and relative conductivity (C) analysis of Alfalfa transgenic with *MsRCI2*s under salt stress.WT stands for Wide-type;A12,A22,B13,B19,C2,C10 were transformedwith the *MsRCI2A,MsRCI2B,* *MsRCI2C* genes respectively.Values are means±SD of three replicates ( Duncan test: *P* < 0.05 ) .
